# The Utility of Inflammatory Serum Markers in the Assessment of Perioperative Morbidity after Radical Cystectomy for Bladder Cancer

**DOI:** 10.3390/medicina59050926

**Published:** 2023-05-11

**Authors:** Francesco Claps, Giulio Rossin, Bas W. G. van Rhijn, Maria Carmen Mir, Laura S. Mertens, Luca Ongaro, Fabio Traunero, Alexandra I. Iachimovsky, Andrea Piasentin, Francesca Vedovo, Alessandro Perotti, Gabriele Tulone, Alessandro Zucchi, Giovanni Liguori, Alchiede Simonato, Riccardo Bartoletti, Carlo Trombetta, Nicola Pavan

**Affiliations:** 1 Urological Clinic, Department of Medicine, Surgery and Health Sciences, University of Trieste, 34149 Trieste, Italy; giulio.rossin93@gmail.com (G.R.); ongarluc@gmail.com (L.O.); fabio.tra92@gmail.com (F.T.); alex.j.iachy@gmail.com (A.I.I.); andrea.piasentin23@gmail.com (A.P.); francesca.vedovo@gmail.com (F.V.); gioliguori33@gmail.com (G.L.);; 2 Department of Urology, Netherlands Cancer Institute-Antoni van Leeuwenhoek Hospital, 1066 CX Amsterdam, The Netherlands; basvanrhijn@hotmail.com (B.W.G.v.R.); ls.mertens@gmail.com (L.S.M.); 3 Department of Urology, Hospital Universitario La Ribera, 46600 Valencia, Spain; mirmare@yahoo.es; 4 Department of Translational Research and New Technologies, University of Pisa, 56126 Pisa, Italy; perotti.alessandro@yahoo.it (A.P.); zucchi.urologia@gmail.com (A.Z.);; 5 Urology Clinic, Department of Surgical, Oncological and Stomatological Sciences, University of Palermo, 90133 Palermo, Italy; gabriele.tulone@gmail.com (G.T.); alchiede@gmail.com (A.S.);

**Keywords:** urinary bladder neoplasms, radical cystectomy, morbidity, postoperative complications, biomarkers

## Abstract

*Background and Objectives*: To date, sparse evidence exists about the impact of inflammatory serum markers in predicting perioperative complications after radical cystectomy (RC) for bladder cancer (BC). Here, we evaluated the role of the neutrophil-to-lymphocyte ratio (NLR), platelet-to-lymphocyte ratio (PLR), lymphocyte-to-monocyte ratio (LMR), systemic immune-inflammation index (SII), C-reactive protein (CRP), and plasma fibrinogen in predicting perioperative morbidity and unplanned 30-days readmission after RC for BC. *Materials and methods*: We relied on a collaborative database of 271 patients who underwent open RC for cT1-4a N0 M0 BC between January 2012 and December 2022. Univariable and multivariable binomial logistic regression analyses were performed to assess the odds ratio (OR) with 95% confidence intervals (CI) testing the ability of each serum marker to predict postoperative complications (any-grade and major complications), and 30-days unplanned readmission. *Results*: The median age at RC was 73 yr (IQR 67–79). A total of 182 (67.2%) patients were male and the median BMI was 25.2 (IQR 23.2–28.4). Overall, 172 (63.5%) patients had a Charlson Comorbidity Index (CCI) greater than 2 points and 98 (36.2%) were current smokers at the time of RC. Overall, 233 (86.0%) patients experienced at least one complication after RC. Of these, 171 (63.1%) patients had minor complications (Clavien–Dindo grade 1–2) while 100 (36.9%) experienced major complications (Clavien–Dindo grade ≥ 3). According to multivariable analysis, current smoking status, high plasma fibrinogen, and preoperative anemia were independently associated with major complications (OR 2.10, 95%CI 1.15–4.90, *p* = 0.02), (OR 1.51, 95%CI 1.26–1.98, *p* = 0.09), and (OR 1.35, 95%CI 1.17–2.57, *p* = 0.03), respectively. Overall, 56 (20.7%) patients experienced a 30-days unplanned readmission. According to univariable analysis, high preoperative CRP and hyperfibrinogenemia were significantly associated with an increased risk of unplanned readmission (OR 2.15, 95%CI 1.15–4.16, *p* = 0.02; OR 2.18, 95%CI 1.13–4.44, *p* = 0.02, respectively). *Conclusions*: In our study, the preoperative immune-inflammation signature described by NLR, PLR, LMR, SII, and CRP showed a low reliability in predicting perioperative course after RC. Preoperative anemia and hyperfibrinogenemia were independent predictors of major complications. Further studies are pending in order to draw definitive conclusions.

## 1. Introduction

Radical cystectomy (RC) and pelvic lymph node dissection (PLND) with or without neoadjuvant chemotherapy (NAC) represent the gold standard treatment for muscle-invasive bladder cancer (MIBC) and Bacillus of Calmette-Guerin (BCG) unresponsive and refractory non-muscle invasive bladder cancer (NMIBC) [1,2]. Despite surgical advances such as the well-established introduction of a minimally invasive approach to RC and urinary diversion (UD), RC is still burdened by non-negligible perioperative morbidity and postoperative mortality [3,4]. Since patients undergoing RC are commonly elderly and frail, the ability to predict complications and create prevention strategies is crucial in the surgical decision-making process in order to optimize treatment outcomes [5]. Most frequently adopted risk assessment tools such as the American Society of Anesthesiologists (ASA) score, Eastern Cooperative Oncology Group (ECOG), performance status (PS), and the Charlson Comorbidity Index (CCI), include surrogates of comorbidities’ burden. Even though such tools have shown a good predictive value on perioperative mortality rates, they have demonstrated only a moderate performance in terms of perioperative morbidity prediction [4,5,6]. In this context, there is a growing interest in preoperative serum immune-inflammatory markers as predictors of perioperative and postoperative morbidity. Although the ability of inflammatory and immune-nutritional serum markers and related nomograms to define BC prognosis has been extensively evaluated together with standard pathological and immunohistochemistry-based predictors [7,8,9,10,11,12], to date only a few studies have explored the reliability of such tools in predicting perioperative morbidity after RC. The inflammatory response appears to be a fundamental driver in the onset and development of malignancies. In a bid to clarify how inflammation could be related to cancer, two possible different pathways have been proposed, with the first being induced by DNA damage, chromosomal instability, and epigenetic changes, and the second being associated with inflammatory signals caused by a secondary source (autoimmune diseases or infections) [13]. With these prerogatives, the cancer microenvironment enriched by cytokines, transcription factors, and infiltrating immune cells, could be able to enhance both the tumor’s growth and its immune escape ability [14]. Such a scenario might exacerbate a catabolic condition induced by the primary BC itself potentially leading to cancer development and progression by promoting tumoral cachexia, which has been proven to be a risk factor for poorer perioperative recovery [15,16]. Thus, the host’s anabolism and immune competence could be impaired and serum marker alterations might mirror such an imbalance. Here, we tested the reliability of standard preoperative serum parameters such as the neutrophil-to-lymphocyte ratio (NLR), platelet-to-lymphocyte ratio (PLR), lymphocyte-to-monocyte ratio (LMR), systemic immune-inflammation index (SII), C-reactive protein (CRP), and fibrinogen in predicting perioperative morbidity after RC.

## 2. Materials and Methods

### 2.1. Patients’ Selection and Variables

We relied on a retrospective collaborative database including 271 non-consecutive patients who underwent open RC, PNLD, and UD for cT1-4aN0M0 BC between January 2012 and December 2022. Demographic, clinicopathological, and perioperative outcomes data were collected. The study was conducted in accordance with the principles outlined in the Declaration of Helsinki and was centrally approved by the ethical institutional review board of the University of Trieste (ID 113/2021).

Preoperatively, all patients underwent routine laboratory assessment, as well as clinical staging with computed tomography of the chest, abdomen, and/or pelvis. Patients with an acute infection or any other acute or chronic systemic inflammatory condition, as well as those harboring other malignancies at the time of surgery, were excluded from the analysis. Variables collected included age, gender, CCI, body mass index (BMI), ASA score, NAC administration, smoking status, history of previous abdominal surgery and/or radiotherapy, length of stay (LOS), perioperative complications, estimated blood loss (EBL), operative time, 30-days readmission, pathological tumor (pT) and nodal (pN) stage, number of lymph nodes (LNs) removed, type of UD, tumor grade, presence of concomitant carcinoma in situ (CIS), lymphovascular invasion (LVI), positive surgical margins (PSMs), presence of variant histologies (VHs), and type of UD.

### 2.2. Endpoints

The endpoints of the current analysis were perioperative complications and unplanned 30-days readmission. We followed the European Association of Urology quality criteria for standardized reporting (Table 1) [17]. Complications were reported according to the Clavien–Dindo classification [18]. Major complications were defined as grade ≥ 3. Any event occurring during the in-hospital stay was considered. Readmission at 30 days was defined as any subsequent and unplanned event occurring within 30 days from the day of discharge of the index hospitalization. The cause of death was extracted from the medical reports and/or from death certificates.

### 2.3. Preoperative Serum Markers Assessment and Cut-Off Determination

Laboratory parameters were routinely measured 30 days before RC; the NLR, PLR, LMR, and SII were calculated, and fibrinogen and CRP values were collected. Patients lacking these data were excluded from the analysis. The calculation algorithm of each marker is presented in Table 2. The NLR, PLR, LMR, and SII were analyzed continuously and further dichotomized according to cut-offs already used in the literature, namely 2.5, 150, 3.41, and 610, respectively [18,19,20,21,22,23,24]. According to laboratory standards, the fibrinogen and CRP values were 350 mg/dL and 5.0 mg/L, respectively. Preoperative anemia was defined according to WHO criteria as haemoglobin levels lower than 12.0 g/dL or 13.0 g/dL for women and men, respectively.

### 2.4. Pathological Evaluation

All RC specimens were locally reviewed by a dedicated uropathologist. The pathological stage was defined according to the 2017 TNM classification system (eighth edition), while the tumoral grade was based on the 2004/2016 WHO system. The pathological review was performed according to the 2016 WHO classification of bladder tumors [25]. Pure non-urothelial VH cases were excluded.

### 2.5. Statistical Analysis

Descriptive analysis included frequencies and proportions for categorical variables. Medians and the interquartile range (IQR) were reported for continuous coded variables. The Chi-square or Fisher’s exact test was used to compare categorical variables. To compare continuous variables, Student’s *t*-test was used when normality could be accepted and the Mann–Whitney U test, conversely. All tests were two-sided with a level of significance set at *p* < 0.05. Univariable and multivariable binomial logistic regression analyses were performed to assess the odds ratio (OR) with 95% confidence intervals (CI) testing the ability of each serum marker to predict postoperative complications (any-grade and major complications), and 30-days unplanned readmission. Significant covariates at univariable analysis were entered into the multivariable model together with non-modifiable preoperative characteristics such as age, gender, CCI, ASA score, BMI, history of preoperative abdominal surgery or radiotherapy, NAC administration, smoking status, and the choice of UD. Data analysis was performed using R language programming (Vienna, Austria: R Foundation for Statistical Computing; 2018, version 3.6.3-https://www.R-project.org/).

## 3. Results

### 3.1. Descriptive Analyses of Clinicopathological and Surgical Characteristics

All patients’ demographics, clinicopathological characteristics, and perioperative outcomes are depicted in Table 3. Our study population comprised 271 patients with a median age of 73 yr (IQR 67–79), 182 (67.2%) patients were male, and the median BMI was 25.2 (IQR23.2–28.4). Overall, 172 (63.5%) patients had a CCI greater than 2 points and 98 (36.2%) were current smokers at the time of RC. The median operative time was 280 min (IQR 240–330), 210 (77.5%) patients underwent RC with ileal conduit diversion, 50 patients (18.5%) received a cutaneous ureterostomy, and 13 patients (4.8%) an orthotopic ileal neobladder. Overall, 144 (53.1%) patients had locally advanced disease and in 69 (25.5%) a nodal involvement was described at the final pathology. The median number of LNs removed was 12 and a VH was found in 79 (29.2%) cases. The median values for the NLR, PLR, LMR, SII, PNI, CONUT score, albumin, fibrinogen, and CRP were 2.8 (IQR 2.0–4.2), 151.9 (IQR 119.6–210.1), 2.6 (IQR 1.8–3.4), 705.8 (IQR 449.3–1090.6), 48.3 (IQR 42.7–52.1), 0 (IQR 1–3), 4.0 g/dl (IQR 3.6–4.3), 390.0 mg/dL (IQR 326.0–482.0), and 6.2 mg/L (IQR 1.8–18.0), respectively. Preoperative anemia was detected in 142 (52.4%) patients.

### 3.2. Prediction of Postoperative Morbidity and 30-Days Readmission

As a whole, 233 (86.0%) patients experienced some complications after RC. Of these, 171 (63.1%) patients had minor complications (grade 1–2) while 100 (36.9%) experienced major complications (grade ≥ 3). The median LOS was 19 days (IQR, 16–25), and perioperative death (Clavien grade 5) occurred in 7 (2.5%) patients (Table 3). Overall, 413 complications were reported with the majority of them being infective in nature and identified in 121 (29.3%) cases. The median number of per-patient perioperative complications was one (IQR 1–8).

Univariable and multivariable logistic regression models are shown in Table 4. According to univariable analysis, both high NLR and low LMR evaluated as continuous covariates approached a borderline significance (OR 1.22, 95%CI 1.02–1.55, *p* = 0.07), and (OR 0.84, 95%CI 0.68–1.04, *p* = 0.09), respectively. The type of UD performed, history of previous abdominal surgery and/or radiotherapy, NAC administration, and smoking status were not associated with an increased risk of any-grade complications after RC.

Considering the occurrence of major complications, according to univariable analysis, high CCI (≥2), current smoking status, high preoperative fibrinogen levels, and preoperative anemia were significantly associated with an increased risk of developing major complications (OR 2.23, 95%CI 1.01–5.73, *p* = 0.04), (OR 2.11, 95%CI 1.22–4.63, *p* = 0.01), (OR 1.65, 95%CI 1.36–3.16, *p* = 0.01), and (OR 1.83, 95%CI 1.60–2.63, *p* = 0.02), respectively. According to multivariable analysis, current smoking status, high fibrinogen, and preoperative anemia were independently associated with the occurrence of major complications, (OR 2.10, 95%CI 1.15–4.90, *p* = 0.02), (OR 1.51, 95% CI1.26–1.98, *p* = 0.03), and (OR 1.35, 95%CI 1.17–2.57, *p* = 0.03). A detailed description of perioperative morbidity is presented in Table 5.

Unplanned readmission at 30 days was reported in 56 (20.7%) patients. According to univariable analysis, high preoperative CRP and hyperfibrinogenemia were significantly associated with an increased risk of unplanned readmission (OR 2.15, 95%CI 1.15–4.16, *p* = 0.02) and (OR 2.18, 95%CI 1.13–4.44, *p* = 0.02), respectively. Statistical significance was not reached for conventional comorbidity assessment tools such as CCI and ASA scores. According to multivariable analysis, none of the examined factors were independently associated with an increased risk of unplanned readmission at 30 days.

## 4. Discussion

In this retrospective multi-institutional experience, we evaluated the role of the preoperative immune-inflammation serum markers in a cohort of BC patients undergoing RC and UD. We found low reliability of these laboratory tools in predicting overall perioperative morbidity and unplanned readmission, whereas the preoperative anemic state and hyperfibrinogenemia were independently associated with the occurrence of major complications.

Both the surgical decision-making process and counseling of patients undergoing RC and UD are complex, while an individualized approach is mandatory to balance the benefits of an extirpative procedure for a life-threatening malignancy and the risk of perioperative morbidity [6]. Studies have shown that CCI [26], ECOG performance status [27], frailty index [28], and ASA score [29] are independent predictors of postoperative complications and mortality in the 90 days following RC.

Similarly to Vetterlein et al., we applied a meticulous assessment of in-hospital stay morbidity. We found that 233 (86.0%) patients experienced at least one complication during the postoperative course highlighting the urgent clinical need for reliable and objective risk assessment tools. A systemic inflammatory response is a crucial factor in cancer patients, and mounting evidence suggests that the inflammatory process plays a key role in promoting proliferation, angiogenesis, invasion, and progression [30]. As a result, markers of systemic inflammation have been extensively incorporated into prognostic models to further refine the survival outcomes prediction of patients with BC undergoing radical treatment [31,32,33,34].

We found no significant predictors in terms of any-grade complications’ occurrence at multivariable analysis. Conversely, preoperative anemia was independently associated with major complications’ occurrence after RC. In the context of BC, anemia development is related to multiple contributing factors such as oncological treatments, malnutrition, and haematuria. On the other hand, blood transfusions could be considered an additive burden, with several reports highlighting the immunosuppressive effect of blood transfusions which could potentially cause a predisposition to postoperative complications and an overall worse prognosis [35]. Carvalho et al. found that anemic patients had increased odds of both minor and major complications after RC [36]. Particularly, anemic patients were affected by a greater risk of developing gastrointestinal, infectious, pulmonary, genitourinary, and renal-related complications. Within a setting where NAC was not administrated, Vetterlein et al. found that delta hemoglobin was one of the main drivers of post-RC complications. Anemia is an objective measure as well as a reversible condition that can be efficaciously prevented and addressed perioperatively. In the context of a randomized clinical trial, Froessler et al. reported a beneficial impact of intravenous iron administration before major abdominal surgeries in patients with iron deficiency anemia in order to reduce the blood transfusions rate and LOS [37].

According to our results, preoperative hyperfibrinogenemia is independently associated with major complications. Fibrinogen is a crucial plasma glycoprotein in the formation of blood clots that plays a key role as an acute phase reactant and represents a prognostic biomarker in cancer progression [38]. The prognostic role of plasma fibrinogen has been evaluated in several urological malignancies [39,40]. Considering perioperative morbidity, Mari et al., in the setting of an elderly population undergoing RC, found results that mirrored ours [41]. This is the second experience reporting such findings. Within a cohort of 694 patients, the authors, after considering all other significant covariates, highlighted an independent impact of hyperfibrinogenemia on major complications’ development after RC. Thus, fibrinogen could serve as a simple and cost-effective marker in the preoperative workup of RC.

Unplanned readmission after RC has an important impact on both survival outcomes and health-related quality of life [42]. However, the introduction of the robotic approach has not yielded a substantial decrease in the rate of readmissions compared to open surgery [43]. In our study, CRP was associated with 30-days readmission in our univariable model. However, after taking into account all the other confounders, significant contributions were not found. Previous evidence has identified the increasing age of patients undergoing RC, comorbidity burden, and intraoperative complications as the primary predictors of unplanned readmission [44,45,46]. Conversely, considering the 90-days readmission frequency among contemporary patients undergoing robot-assisted RC with intracorporeal diversion, Cacciamani et al. found an overall unplanned readmission rate of 29% with infective complications in the index hospitalization representing the main predictor. Particularly, no impact of conventional variables such as age, gender, ASA score, type of UD, and BMI was described.

Our study is not devoid of limitations that are associated with the retrospective design. The application of Receiver Operating Characteristic (ROC) Curve analysis would have been the ideal statistical method in this specific scenario together with the development of both discovery and validation cohorts. Moreover, it has to be acknowledged that, despite the multi-institutional setting, the small sample size was one of the main limitations of the current analysis. In addition, the low rate (9.2%) of standard-of-care NAC administration represents a concern. At each institution, all the patients received the enhanced recovery after surgery (ERAS) protocol. However, due to the lack of standardized measures it was not included at the time of the regression models’ development. Reasons for unplanned readmission were not always available and we did not consider the surgeons’ caseload as a possible predictor. Nevertheless, this is one of the few multicenter experiences that evaluated the impact of immune-inflammatory serum markers on the perioperative course of RC patients adhering to the European Association of Urology quality criteria for standardized reporting of complications.

## 5. Conclusions

The preoperative immune-inflammatory status as described by NLR, PLR, LMR, SII, and CRP demonstrated a low reliability in predicting perioperative course after RC. Preoperative anemia and hyperfibrinogenemia were independent predictors of major complications. Being easy-to-use and inexpensive, in the future these laboratory markers could become part of a more refined risk-stratification system of RC candidates. Further studies are pending in order to draw definitive conclusions.

## Figures and Tables

**Table 1:** European Association of Urology quality criteria for comprehensive reporting of surgical outcomes after RC and their implementation [17].

	EAU Quality Criteria	Implementation
1	Define the method of accruing data	Retrospective analysis of prospectively maintained data of digitalized medical charts
2	Define who collected the data	Residents in Urology
3	Indicate the duration of follow-up	Within 90 days after RC and UD
4	Include outpatient information	Outpatient information was included
5	Include mortality data and causes of death	Mortality and causes of death were reported
6	Include definitions of complications	A predefined complication catalog including definitions of general- and procedure-specific complications was reported
7	Define procedure-specific complications	
8	Report intra- and postoperative complications separately	Intraoperative and postoperative complications were separately described
9	Use a severity grading system for postoperative complications (avoiding the distinction minor/major)	Clavien–Dindo classification
10	Postoperative complications should be presented in a table either by grade or by complication type (specific grades should always be provided; grouping is not accepted)	A detailed table of postoperative complications, including grading, treatment, and proportions was provided
11	Include risk factors	The CCI and the ASA score were included in the analyses. Other clinical variables such as smoking status, previous abdominal surgery, and previous radiotherapy on abdomen and/or pelvis were included
12	Include readmissions and causes	None.
13	Include reoperations, types, and causes	Reoperations, types, and causes were tabulated.
14	Include the percentage of patients lost to follow-up	No patients were lost on follow-up.

Abbreviations are as follows: EAU: European Association of Urology; RC: Radical Cystectomy; UD: Urinary Diversion.

**Table 2:** Serum markers’ definition.

Serum Marker	Formula	Cut-Off
NLR	neutrophil count (10^9^/L)/lymphocyte count (10^9^/L)	2.5
PLR	platelet count (10^9^/L)/lymphocyte count (10^9^/L)	150
LMR	lymphocyte count (10^9^/L)/monocyte count (10^9^/L)	3.4
SII	[neutrophil count (10^9^/L) × platelet count (10^9^/L)]/lymphocyte count (10^9^/L)	610
Fibrinogen	-	350 mg/dL
CRP	-	5.0 mg/L

Abbreviations are as follows: NLR: neutrophil-to-lymphocyte ratio; PLR: platelet-to-lymphocyte ratio; LMR: lymphocyte-to-monocyte ratio; SII: systemic immune-inflammation index; CRP: C-reactive protein.

**Table 3:** Descriptive baseline clinicopathological data and perioperative findings of 271 non-consecutive patients with BC treated with RC and PLND.

Variables	Overall
Patients, n. (%)	271 (100.0)
Age (years), median (IQR)	73 (67–79)
Gender, n. (%)	
Male	182 (67.2)
Female	89 (32.8)
BMI, median (IQR)	25.2 (23.2–28.4)
CCI, n. (%)	
0	52 (19.2)
1	47 (17.3)
≥2	172 (63.5)
ASA score	
1, 2	128 (47.2)
3, 4	143 (52.8)
Smoking status, n. (%)	
Never	82 (30.3)
Current	98 (36.2)
Former	91 (33.6)
Previous abdominal surgery, n. (%)	123 (45.4)
Previous radiotherapy on abdomen or pelvis, n. (%)	14 (5.2)
NLR, median (IQR)	2.8 (2.0–4.2)
PLR, median (IQR)	151.9 (119.6–210.1)
LMR, median (IQR)	2.6 (1.8–3.4)
SII, median (IQR)	705.8 (449.3–1090.6)
Fibrinogen (mg/dL), median (IQR)	390.0 (326.0–482.0)
CRP (mg/L), median (IQR)	6.2 (1.8–18.0)
Neoadjuvant chemotherapy, n. (%)	25 (9.2)
Preoperative anemia, n. (%)	142 (52.4)
Operative time (minutes), median (IQR)	280 (240–330)
Estimated blood loss (ml), median (IQR)	500 (350–700)
Intraoperative blood transfusions, n. (%)	42 (15.5)
Urinary diversion, n. (%)	
Ileal Conduit	210 (77.5)
Orthotopic Neobladder	13 (4.8)
Ureterocutaneostomy	50 (18.5)
pT-stage, n. (%)	
pT0	27 (10.0)
NMIBC (pTa/is/1)	40 (14.8)
pT2	60 (22.1)
pT3	93 (34.3)
pT4	51 (18.8)
pN-stage, n. (%)	
pN negative	202 (74.5)
pN positive	69 (25.5)
N. of lymph nodes removed, median (IQR)	12 (8–19)
Concomitant CIS, n. (%)	34 (12.5)
High Tumor Grade, n. (%)	214 (79.0)
Variant Histology, n. (%)	79 (29.2)
LVI, n. (%)	127 (46.9)
PSMs, n. (%)	41 (15.1)
Clavien complication grade, n. (%)	
None	38 (14.0)
1	60 (22.1)
2	111 (41.0)
3 (a, b)	39 (14.4)
4 (a, b)	16 (5.9)
5	7 (2.5)
Complications experienced per patient, median (range)	1 (1–8)
Length of stay (days), median (IQR)	19 (16–25)
30-days readmission, n. (%)	56 (20.7)

Abbreviations are as follows: BC: bladder cancer; RC: radical cystectomy; PLND: pelvic lymph node dissection; CONUT: Controlling Nutritional Status; IQR: interquartile range; BMI: body mass index; CCI: Charlson comorbidity index; ASA: American Society of Anaesthesiologists; NLR: neutrophil to lymphocyte ratio; PLR: platelet to lymphocyte ratio; LMR: lymphocyte to monocyte ratio; SII: systemic immune-inflammation index; PNI: Prognostic Nutritional Index; CRP: C-reactive protein. pT-stage: pathological tumor stage; pN-stage: pathological nodal stage; CIS: carcinoma in situ; LVI: lymphovascular invasion; PSMs: positive surgical margins; VHs: variant histologies.

**Table 4:** Univariable and Multivariable logistic regression analyses for prediction of overall postoperative complications, major postoperative complications, and 30-days readmission among 271 patients with clinical localized BC treated with RC and PLND.

	Any Grade Complications (1–5)				Major Complications (3–5)				30-Days Readmission			
	Univariable		Multivariable		Univariable		Multivariable		Univariable		Multivariable	
Variable	OR 95% CI	*p*	OR 95% CI	*p*	OR 95% CI	*p*	OR 95% CI	*p*	OR 95% CI	*p*	OR 95% CI	*p*
Age (as cont.)	1.03 (0.99–1.07)	0.11	1.02 (0.98–1.07)	0.3	1.02 (0.98–1.05)	0.4	1.01 (0.97–1.05)	0.5	1.00 (0.97–1.04)	0.8	1.00 (0.97–1.04)	0.8
Sex												
Female	1.00 (Ref.)	-	1.00 (Ref.)	-	1.00 (Ref.)	-	1.00 (Ref.)	-	1.00 (Ref.)	-	1.00 (Ref.)	-
Male	0.59 (0.25–1.26)	0.2	0.54 (0.22–1.21)	0.2	1.51 (0.81–2.92)	0.2	1.40 (0.72–2.82)	0.7	0.64 (0.35–1.18)	0.15	0.73 (0.38–1.44)	0.4
BMI (as cont.)	1.06 (0.97–1.16)	0.2	0.99 (0.92–1.07)	0.8	1.02 (0.95–1.09)	0.6	1.03 (0.92–1.11)	0.8	1.05 (0.97–1.13)	0.2	0.98 (0.77–1.34)	0.3
CCI												
0	1.00 (Ref.)	-	1.00 (Ref.)	-	1.00 (Ref.)	-	1.00 (Ref.)	-	1.00 (Ref.)	-	1.00 (Ref.)	-
1	2.88 (0.91–11.1)	0.09	1.82 (0.74–4.61)	0.2	1.74 (0.61–5.21)	0.2	1.11 (0.34–3.68)	0.5	0.47 (0.16–1.27)	0.15	0.38 (0.11–1.20)	0.11
≥2	1.73 (0.75–3.77)	0.2	1.52 (0.62–3.80)	0.4	2.23 (1.01–5.73)	0.04	1.31 (0.52–3.62)	0.6	0.70 (0.35–1.46)	0.3	0.58 (0.23–1.46)	0.2
ASA score												
1, 2	1.00 (Ref.)	-	1.00 (Ref.)	-	1.00 (Ref.)	-	1.00 (Ref.)	-	1.00 (Ref.)	-	1.00 (Ref.)	-
3, 4	1.44 (0.73–2.91)	0.1	1.02 (0.45–2.29)	0.9	1.53 (0.86–2.76)	0.2	1.28 (0.66–2.51)	0.2	1.05 (0.58–1.90)	0.9	1.17 (0.58–2.42)	0.7
NAC												
No	1.00 (Ref)	-	1.00 (Ref)	-	1.00 (Ref)	-	1.00 (Ref.)	-	1.00 (Ref.)	-	1.00 (Ref.)	-
Yes	0.85 (0.30–3.03)	0.8	1.16 (0.38–4.42)	0.8	0.84 (0.27–2.19)	0.8	0.71 (0.21–2.06)	0.6	0.49 (0.11–1.50)	0.3	0.84 (0.18–2.81)	0.8
Previous abdominal surg.												
No	1.00 (Ref.)	-	1.00 (Ref.)	-	1.00 (Ref.)	-	1.00 (Ref.)	-	1.00 (Ref.)	-	1.00 (Ref.)	-
Yes	1.11 (0.55–2.27)	0.8	1.14 (0.54–2.47)	0.7	1.02 (0.57–1.81)	0.9	0.93 (0.49–1.73)	0.7	0.96 (0.53–1.73)	0.9	1.04 (0.54–2.01)	0.8
Previous abdominal RT.												
No	1.00 (Ref.)	-	1.00 (Ref.)	-	1.00 (Ref.)	-	1.00 (Ref.)	-	1.00 (Ref.)	-	1.00 (Ref.)	-
Yes	0.96 (0.25–6.33)	0.9	0.79 (0.18–5.52)	0.8	0.92 (0.20–3.07)	0.9	1.07 (0.21–4.12)	0.9	0.62 (0.09–2.35)	0.6	0.56 (0.08–2.40)	0.5
Smoking status												
Never	1.00 (Ref.)	-	1.00 (Ref.)	-	1.00	-	1.00	-	1.00 (Ref.)	-	1.00 (Ref.)	-
Current	1.92 (0.84–4.51)	0.13	1.88 (0.50–8.16)	0.8	2.11 (1.22–4.63)	0.01	2.10 (1.15–4.90)	0.02	0.80 (0.39–1.64)	0.5	1.02 (0.45–2.34)	0.6
Former	1.74 (0.76–4.11)	0.2	1.21 (0.45–3.08)	0.7	2.01 (0.94, 4.46)	0.08	1.92 (0.86–4.46)	0.12	0.83 (0.40–1.72)	0.6	0.70 (0.35–1.46)	0.7
NLR (as cont.)	1.22 (1.02–1.55)	0.07	-	-	1.07 (0.98–1.17)	0.12	-	-	1.05 (0.96–1.15)	0.3	-	-
NLR												
Normal	1.00 (Ref.)	-	-	-	1.00 (Ref.)	-	-	-	1.00 (Ref.)	-	-	-
High	1.47 (0.74–2.93)	0.3			1.64 (0.91–3.04)	0.2			1.69 (0.92–3.20)	0.1		
PLR (as cont.)			1.00 (1.00–1.01)	0.4	1.00 (1.00–1.00)	0.2	-	-	1.00 (1.00–1.00)	0.4	-	-
PLR												
Normal	1.00 (Ref.)	-	-	-	1.00 (Ref.)	-	-	-	1.00 (Ref.)	-	-	-
High	0.69 (0.34–1.37)	0.3		-	0.99 (0.56–1.76)	0.9	-	-	1.05 (0.58–1.90)	0.9	-	-
LMR (as cont.)	0.84 (0.68–1.04)	0.09	-	-	0.91 (0.73–1.10)	0.4	-	-	0.82 (0.64–1.03)	0.1	-	-
LMR												
Normal	1.00 (Ref.)	-	-	-	1.00 (Ref.)	-	-	-	1.00 (Ref.)	-	-	-
Low	1.83 (0.87–3.75)	0.1		-	0.98 (0.52–1.92)	0.9	-	-	1.36 (0.69–2.86)	0.4	-	-
SII (as cont.)	1.00 (1.00–1.00)	0.12	-	-	1.00 (1.00–1.00)	0.2	-	-	1.00 (1.00–1.03)	0.8	-	-
SII												
Normal	1.00 (Ref.)	-	-	-	1.00 (Ref.)	-	-	-	1.00 (Ref.)	-	-	-
High	0.83 (0.40–1.66)	0.6			1.18 (0.66–2.14)	0.11			1.44 (0.79–2.71)	0.11		
CRP												
Normal	1.00 (Ref.)	-	-	-	1.00 (Ref.)	-	-	-	1.00 (Ref.)	-	1.00 (Ref.)	-
High	1.80 (0.89–3.74)	0.11			1.52 (0.84–2.81)	0.2			2.15 (1.15–4.16)	0.02	1.45 (0.69–3.12)	0.2
Fibrinogen												
Normal	1.00 (Ref.)	-	-	-	1.00 (Ref.)	-	1.00 (Ref.)	-	1.00 (Ref.)	-	1.00 (Ref.)	
High	1.33 (0.65–2.66)	0.4			1.65 (1.36–3.16)	0.01	1.51 (1.26–1.98)	0.03	2.18 (1.13–4.44)	0.02	1.70 (0.78–3.86)	
Preoperative anemia												
No	1.00 (Ref.)	-	-	-	1.00 (Ref.)	-	1.00 (Ref.)	-	1.00 (Ref.)	-	-	-
Yes	1.11 (0.56–2.21)	0.8			1.83 (1.60–2.63)	0.02	1.35 (1.17–2.57)	0.03	0.98 (0.54–1.77)	0.7		
Urinary Diversion												
Ureterocuteneostomy	1.00 (Ref.)	-	1.00 (Ref.)	-	1.00 (Ref.)	-	1.00 (Ref.)	-	1.00 (Ref.)	-	1.00 (Ref.)	-
Ileal Conduit	0.61 (0.20–1.52)	0.3	0.69 (0.18–1.76)	0.4	1.04 (0.51–2.28)	0.8	1.04 (0.41–2.89)	0.9	0.93 (0.45–2.04)	0.6	1.18 (0.52–2.80)	0.7
Orthotopic Neobladder	1.33 (0.19–26.8)	0.8	1.20 (0.12–29.9)	0.9	1.06 (0.21–4.23)	0.8	1.09 (0.27–4.73)	0.9	0.64 (0.09–2.89)	0.8	1.09 (0.14–5.88)	0.9

Abbreviations are as follows: BC: bladder cancer; RC: radical cystectomy; PLND: pelvic lymph node dissection; OR: odds ratio; CI: confidence interval; BMI: body mass index; CCI: Charlson comorbidity index; ASA: American Society of Anaesthesiologists; NAC: neoadjuvant chemotherapy; RT: radiotherapy; NLR: neutrophil to lymphocyte ratio; PLR: platelet to lymphocyte ratio; LMR: lymphocyte to monocyte ratio; SII: systemic immune-inflammation index; CRP: C-reactive protein; UD: urinary diversion; pT-stage: pathological tumor stage; pN-stage: pathological nodal stage; CONUT: Controlling Nutritional Status.

**Table 5:** Detailed description of in-hospital stay complications occurred in 271 patients who underwent open RC, PLND, and UD.

Type of Complications	N. (%)
Gastrointestinal	86 (20.8)
Paralytic Ileus	17
Mechanical Ileus	9
Bowel perforation	3
Clostridium difficile colitis	2
Gastrointestinal bleeding	3
Emesis	37
Infectious	121 (29.3)
Fever of unknown origin	35
Bacteriuria (>105 CFU/mL; asymptomatic)	14
Urinary tract infection (>105 CFU/mL; symptomatic)	12
Abscess	12
Sepsis (SIRS in response to infectious process)	39
Septic shock	2
Pyelonephritis	2
Gastroenteritis	3
Pancreatitis	2
Wound	47 (11.4)
Wound dehiscence	31
Wound infection	16
Genitourinary	21 (5.1)
Acute kidney injury	8
Parastomal hernia	1
Ureteral stricture	4
Urinary fistula	7
Anastomotic stricture	1
Cardiac	13 (3.1)
Arrhythmia (atrial fibrillation)	4
Myocardial infarction	6
Hypertension (new onset)	1
(Acute) congestive heart failure	2
Pulmonary	36 (8.7)
Pneuomonia	21
Bronchitis	2
Respiratory distress/dyspnea	7
Pleural effusion	6
Bleeding	62 (15.0)
Anemia with transfusion of blood products	54
Anemia with adoption administration of iron-derived products	6
Hematoma	2
Thromboembolic	10 (2.4)
Deep vein thrombosis	2
Pulmonary embolism	8
Neurological	11 (2.7)
Peripheral neuropathy	4
CVA/TIA	1
Delirium	3
Loss of consciousness/syncope	3
Miscellaneous	6 (1.5)
Acidosis	2
Lymphocele	2
Catheter dislocation (Ureteral, Suprapubic, transurethral)	2
Cumulative complications	413 (100.0)

Abbreviations are as follows RC: radical cystectomy; PLND: pelvic lymph node dissection; UD: urinary diversion; CVA: cerebrovascular accident; TIA: transient ischemic attack.

## Data Availability

Not applicable.
